# Supplementary material for: Nef mediates neuroimmune response, myelin impairment, and neuronal injury in EcoHIV-infected mice
Source: Life Sci Alliance. 2024 Nov 12;8(2):e202402879. doi: 10.26508/lsa.202402879 (PMC11557684; doi:10.26508/lsa.202402879)
Supplement: Supplementary file 1 [file LSA-2024-02879_TableS1.docx]

**Supplemental Table S1. Primers* used for PCR.**

| **Target Gene** | **GenBank Accession #** | **Sequence of the Primers** |
| --- | --- | --- |
| ABCA1 | NM_013454 | F: GGAGCCTTTGTGGAACTCTTCC  R: CGCTCTCTTCAGCCACTTTGAG |
| AIF1 (IBA1) | NM_019467 | F: TCTGCCGTCCAAACTTGAAGCC  R: CTCTTCAGCTCTAGGTGGGTCT |
| Beta Actin | NM_007393 | F: CATTGCTGACAGGATGCAGAAGG  R: TGCTGGAAGGTGGACAGTGAGG |
| C3 | NM_009778 | F: CGCAACGAACAGGTGGAGATCA  R: CTGGAAGTAGCGATTCTTGGCG |
| CCL2 (MCP-1) | NM_011333 | F: GCTACAAGAGGATCACCAGCAG  R: GTCTGGACCCATTCCTTCTTGG |
| CCL3 (MIP-1a) | NM_011337 | F: ACTGCCTGCTGCTTCTCCTACA  R: ATGACACCTGGCTGGGAGCAAA |
| CxCl10 (IP10) | NM_021274 | F: ATCATCCCTGCGAGCCTATCCT  R: GACCTTTTTTGGCTAAACGCTTTC |
| IFNγ | NM_008337 | F: CAGCAACAGCAAGGCGAAAAAGG  R: TTTCCGCTTCCTGAGGCTGGAT |
| IL-1β | NM_008361 | F: TGGACCTTCCAGGATGAGGACA  R: GTTCATCTCGGAGCCTGTAGTG |
| IL-6 | NM_031168 | F: TACCACTTCACAAGTCGGAGGC  R: CTGCAAGTGCATCATCGTTGTTC |
| STAT1 | NM_009283 | F: GCCTCTCATTGTCACCGAAGAAC  R: TGGCTGACGTTGGAGATCACCA |
| TNF-α | NM_013693 | F: GGTGCCTATGTCTCAGCCTCTT  R: GCCATAGAACTGATGAGAGGGAG |
| TREM2 | NM_031254 | F: CTACCAGTGTCAGAGTCTCCGA  R: CCTCGAAACTCGATGACTCCTC |

*Primers purchased from OriGene.
