# Supplementary material for: Nef mediates neuroimmune response, myelin impairment, and neuronal injury in EcoHIV-infected mice
Source: Life Sci Alliance. 2024 Nov 12;8(2):e202402879. doi: 10.26508/lsa.202402879 (PMC11557684; doi:10.26508/lsa.202402879)
Supplement: Supplementary file 2 [file LSA-2024-02879_TableS2.docx]

**Supplemental Table S2. Comparative quantification cycle (ΔΔC_q_) calculation method for qPCR.**

| **Given Values** | **Step 1** | **Step 2** | **Step 3** | **Step 4** |
| --- | --- | --- | --- | --- |
| C_q_^(REF)^ & C_q_^(TARG)^ | ∆C_q_  = C_q_^(TARG)^ – C_q_^(REF)^ | ∆C_q_ Expression  = 2–∆C_q_ | Average replicates and calculate standard deviation | ∆∆C_q_  =Normalize to treatment control |

Adapted from Horizon Discovery technical note.
