# Supplementary material for: Nef mediates neuroimmune response, myelin impairment, and neuronal injury in EcoHIV-infected mice
Source: Life Sci Alliance. 2024 Nov 12;8(2):e202402879. doi: 10.26508/lsa.202402879 (PMC11557684; doi:10.26508/lsa.202402879)
Supplement: Supplementary file 3 [file LSA-2024-02879_TableS3.docx]

**Supplemental Table S3. List of antibodies used for immunofluorescence.**

| **Antibodies** | **Vendor** | **Cat. #** | **Clone** | **Dilution** | **Use** |
| --- | --- | --- | --- | --- | --- |
| Rabbit anti-microtubule-associated protein 2 (MAP2) | EMD Millipore | AB5622 | polyclonal | 1:150 | detection of dendrites |
| Rabbit anti- ionized calcium-binding adapter molecule 1 (IBA1) | Wako Chemicals | 019-19741 | polyclonal | 1:500 | detection of microglia/ myeloid cells |
| Rat anti- glial fibrillary acidic protein (GFAP) | Invitrogen | 13-0300 | 2.2B10 | 1:500 | detection of astrocytes |
| Mouse anti-myelin basic protein (MBP) | BioLegend | 836504 | SMI-99 | 1:500 | detection of myelin |
| Goat anti-rabbit Alexa 594 | Invitrogen | A11012 | – | 1:500 | secondary |
| Goat anti-mouse Alexa 594 | Invitrogen | A11032 | – | 1:500 | Secondary |
| Goat anti-rat Alexa 488 | Invitrogen | A11006 | – | 1:500 | secondary |
| Goat anti-rabbit Alexa 488 | Invitrogen | A11008 | – | 1:500 | secondary |
